# IL2RA/CD25 Gene Polymorphisms: Uneven Association with Multiple Sclerosis (MS) and Type 1 Diabetes (T1D)

**DOI:** 10.1371/journal.pone.0004137

**Published:** 2009-01-06

**Authors:** Antonio Alcina, María Fedetz, Dorothy Ndagire, Oscar Fernández, Laura Leyva, Miguel Guerrero, María M. Abad-Grau, Carmen Arnal, Concepción Delgado, Miguel Lucas, Guillermo Izquierdo, Fuencisla Matesanz

**Affiliations:** 1 Instituto de Parasitología y Biomedicina López Neyra, Consejo Superior de Investigaciones Científicas, Granada, Spain; 2 Servicio de Neurología, Instituto de Neurociencias Clínicas, Hospital Carlos Haya, Málaga, Spain; 3 Departamento de Lenguajes y Sistemas Informáticos, Universidad de Granada, Granada, Spain; 4 Servicio de Neurología, Hospital Virgen de las Nieves, Granada, Spain; 5 Centro Regional de Transfusión Sanguínea Granada-Almería, Granada, Spain; 6 Servicio de Biología Molecular, Hospital Virgen Macarena, Sevilla, Spain; 7 Unidad de Esclerosis Múltiple, Hospital Virgen Macarena, Sevilla, Spain; Peninsula Medical School, United Kingdom

## Abstract

**Background:**

IL-2 receptor (IL2R) alpha is the specific component of the high affinity IL2R system involved in the immune response and in the control of autoimmunity.

**Methods and Results:**

Here we perform a replication and fine mapping of the *IL2RA* gene region analyzing 3 SNPs previously associated with multiple sclerosis (MS) and 5 SNPs associated with type 1 diabetes (T1D) in a collection of 798 MS patients and 927 matched Caucasian controls from the south of Spain. We observed association with MS in 6 of 8 SNPs. The rs1570538, at the 3′- UTR extreme of the gene, previously reported to have a weak association with MS, is replicated here (P = 0.032). The most associated T1D SNP (rs41295061) was not associated with MS in the present study. However, the rs35285258, belonging to another independent group of SNPs associated with T1D, showed the maximal association in this study but different risk allele. We replicated the association of only one (rs2104286) of the two *IL2RA* SNPs identified in the recently performed genome-wide association study of MS.

**Conclusions:**

These findings confirm and extend the association of this gene with MS and reveal a genetic heterogeneity of the associated polymorphisms and risk alleles between MS and T1D suggesting different immunopathological roles of IL2RA in these two diseases.

## Introduction

Multiple sclerosis (MS) is the most common central nervous system disease in young adults, and one of the leading causes of disability in this age group affecting over 2.5 million individuals world-wide [1] The prevalence and incidence rates in Spain are around 77/100 000 habitants and 5.3/100 000 habitants per year respectively similar to what has been found in Britain [2], [3] The disorder, which is presumed to be autoimmune in nature, is characterized by inflammation and demyelination, with axonal and neuronal degeneration. Susceptibility to MS is thought to be conferred by the combination of many common gene variants (not aberrant gene products) and environmental factors, which are mostly unknown [1], [4].

The most strongly associated region implicated in predisposition to MS is the major histocompatibility complex (MHC) on chromosome 6p21, specifically the HLA-DRB1* 1501 class II allele; but, this account for less than 50% of MS genetics [1], [5]. Recently, other regions have been implicated in MS susceptibility and replicated in different independent populations as the interleukin 7 receptor alpha (*IL7RA*) [6]–[8], the interferon regulatory factor 5 (*IRF5*) gene [9] and the interleukin-2 receptor alpha (*IL2RA*) [8], [10], [11]. The *IL2RA* gene has also been associated with type 1 diabetes (T1D) [12]–[14] and localized the association region in two independent groups of SNPs, spanning overlapping regions of 14 and 40 Kb encompassing *IL2RA* intron 1 and the 5′ regions of *IL2RA* and the RNA binding motif protein 17 (*RBM17*) genes.

Diverse autoimmune diseases may coexist in the same individual and in families, suggesting they might share common susceptibility gene variants implying a common etiology [15], [16]. For example, in families with systemic lupus erythematosus (SLE), other autoimmune mediated diseases, such as MS and rheumatoid arthritis (RA) [17], or families with T1D and MS in Sardinian population [18], occur more frequently than in the general population. Such observations and others suggest the existence of shared genes or involvement of common biochemical pathways in these diseases. This hypothesis is supported by numbers of reports on genes that are associated with more than one autoimmune disease, for example the Protein Tyrosine Phosphatase Nonreceptor 22 (PTPN22) has been associated with T1D [19], RA [20], and SLE [21], the IRF5 with SLE [22], inflammatory bowel diseases [23], RA [24] and MS [9], the FCRL3 with RA, autoimmune thyroid disease, SLE [25] and MS [26], among several other examples. However, population-based studies of >30 000 MS first-degree relatives found no increase in autoimmune disease [27].

As the associated SNPs analyzed in a genome wide association scan (GWAS) [8], candidate-gene association study in MS [10] and T1D [13] were not the same [28] we considered interesting to test if the TID-associated SNPs in the *IL2RA* region [13] were also associated with multiple sclerosis (MS) in our cohort of MS patients. In addition, we confirmed one of the two polymorphisms that have been associated with MS in the GWAS [8] and the one associated with MS in our previous candidate-gene association study of *IL2RA* gene [10].

## Methods

### Study subjects

Case samples comprised 798 patients with clinically defined MS according to Poser's criteria [29] They were obtained from four public hospitals: the Hospital Clínico in Granada (n = 130), the Hospital Virgen de las Nieves de Granada (n = 153), the Hospital Carlos Haya in Málaga (n = 357) and the Hospital Virgen de la Macarena in Seville (n = 158) all three cities within a 200 km radius in the South of Spain. The mean age at the sample collection of the cases was 36 years and mean age of controls at interview was 38 years. The percentage of females was 68% for cases and 59% for controls. All of them were classified as RR (relapsing-remitting) or SP (secondary progressive) MS cases. Controls were 927 blood donors with no history of inflammatory disease attending the blood banks of Granada (n = 619), Seville (n = 138) and Málaga (n = 170). The study was approved by the Ethics Committees of each of the hospitals participating in the study and written informed consent was obtained from all participants.

### Genotyping

The SNPs were selected for being associated with MS (rs1570538, rs2104286, rs12722489) [8], [10] or with T1D (rs10795791, rs4147359, rs7090530, rs41295061, rs35285258) [13] in previous studies.

**Figure 1:**
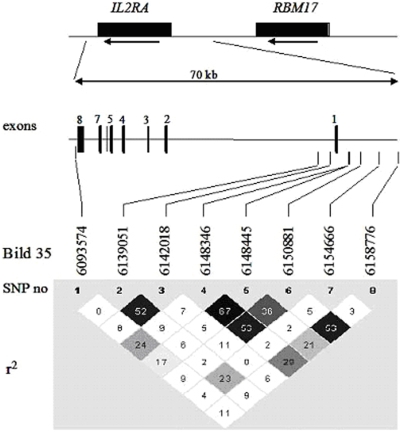
Schematic illustration of *IL2RA* locus showing the positions of the polymorphisms based on the NCBI Bild 35 and the linkage disequilibrium (LD) structure of the *IL2RA* gene as pairwise r^2^-values. The rs41295061 and the rs35285258 in this study correspond to ss52580101 and ss52580135, respectively in the T1D study. ^13^ rs35285258 is in complete LD with rs11594656 (r^2^ = 1) from the T1D study. ^13^ Both ss (submitted snp) numbers can be found as rs (reference snp) numbers in the SNPdb of NCBI.

High-molecular-weight DNA was isolated from whole blood using the Flexigene Kit (Qiagen, Hildren, Gemany) according to the manufacturer's protocol. We studied 8 SNPs located in the IL2RA locus as has been indicated in Fig. 1. rs1570538 was genotyped by restriction fragment length polymorphism method Primer sequences used were as follows: forward, TCATGTGACATCTGGAGGGTTA and reverse, AAAATGAATTTCGTCAATTCGAG, restriction enzyme MwoI. The rest of the SNPs were genotyped by TaqMan technology under conditions recommended by the manufacturer (Applied Biosystems, Foster City, CA, USA). A total of 10 negative controls and 8 duplicates were included for each SNP as a quality control measure.

### Statistical Analysis

The statistical studies to compare allelic and genotypic distribution between patients and controls were performed by Pearson *χ* ^2^ test or Fisher's exact test on 2×2 and 2×3 contingency tables, respectively, using the package available from the web of the Institute of Human Genetics of the Technical University of Munich (http://ihg.gsf.de/cgi-bin/hw/hwa1.pl) and the SPSS 15.0 statistical package. Hardy Weinberg equilibrium was tested using *χ* ^2^ goodness-of-fit test and no significant deviation was observed for any of the SNPs in this study (all P>0.05). Logistic regression analysis was used to calculate odds ratios (ORs) and 95% Confidence Intervals (C.I.s) for genotypes. To evaluate whether the best model is statistically significant, a permutation test was applied to obtain a *p*-value. The Haploview v.3.3 software [30] was used to generate haplotypes, perform haplotype association analysis, and to determine linkage disequilibrium (LD) measures (r^2^) between the polymorphisms. P-values reported are unadjusted except for the most associated SNPs in whose case they were corrected for multiple testing assuming 8 independent SNP markers.

Assuming a minor allele frequency between 6.8–48.4% and a 0.05% prevalence of MS in the Spanish population, we estimated that at the 5% significance level, this study had between 44–92% power to detect genotypic relative risks reported in the first association study. The power that we had to detect effects was actually lower than in the original MS and T1D studies where larger cohorts were used (Table 1).

**Table 1:** Allele distribution and frequency of *IL2RA* SNPs in patients with multiple sclerosis and healthy controls.

SNP	Alleles, n (%)			OR minor allele [95% CI]	P-value		
		Cases	Controls		This work^a^	GWAS/MS^b^	T1D^c^
rs1570538^d^	C	734 (47.8)	851 (51.6)	0.86 [0.75–0.99]	0.033/0.04^d^		
SNP1	T	802 (52.2)	799 (48.4)				
rs2104286	T	1237 (82.3)	1426 (79.1)	0.81 [0.68–0.96]	0.017	2.16×10^−7^	
SNP2	C	265 (17.7)	378 (20.9)				
rs12722489	G	1216 (88.6)	1186(87.2)	0.87 [0.69–1.10]	0.278	2.96×10^−8^	0.52
SNP3	A	156 (11.4)	1742(12.7)				
rs10795791	A	933 (64.4)	1094 (60.6)	0.85 [0.74–0.98]	0.027		1.4×10^−6^
SNP4	G	515 (35.6)	710 (39.4)				
rs4147359	G	1105 (72.6)	1228 (69.0)	0.84 [0.72–0.98]	0.023		1.7×10^−5^
SNP5	A	417 (27.4)	552 (31.0)				
rs7090530^e^	A	741 (48.9)	996 (54.2)	1.23 [1.07–1.41]	0.003		3.6×10^−10^
SNP6	C	767 (51.0)	840 (45.8)				
rs41295061^f^	C	1436 (94.5)	1605 (93.2)	0.80 [0.60–1.07]	0.135		2.8×10^−12^
SNP7	A	84 (5.5)	117 (6.8)				
rs35285258^e^^, ^ g	C	921 (62.4)	1193 (67.7)	1.26 [1.09–1.46]	0.0016		5.3×10^−4^
SNP8	T	555 (37.6)	569 (32.3)				

a P-values = Pearson's goodness-of-fit chi-square (df = 1).

b GWAS/MS combined analysis including 1540 family trios, 2322 case subjects, and 5418 control subjects.^8^

c T1D study from 2965 cases and 2548 controls [13].

d Data from our previous work [10]. IL2RA/MS association study from 346 cases and 413 controls included in the present study.

e rs7090530 and rs35285258 survived Bonferroni correction.

f rs41295061 named ss52580101 in T1D study [13].

g rs35285258 named ss52580135 in T1D study [13].

## Results

Eight polymorphisms in the *IL2RA* gene were genotyped in 798 MS patient samples and 927 sex-matched Caucasian controls from the south of Spain. The linkage disequilibrium (LD) and their precise localization in the chromosome are indicated in the Fig. 1. The rs1570538 is located at the 3′-untranslated region (3′- UTR) of *IL2RA* [10], rs2104286 and rs12722489 are located at the 5′-proximal intron 1 region of the *IL2RA* [8], and the SNPs rs10795791, rs4147359, rs7090530, rs41295061 and rs35285258 are located at the 5′-upstream region of the *IL2RA*, in the intergenic region between *IL2RA* and *RBM17* genes [13].

We found the additive effect of allele dosage as the most plausible genetic model for rs2104286, rs7090530 and rs35285258. The recessive model seemed to fit better for rs1570538 (Table S1). The best model for all the other SNPs was also the additive one among the four tried: additive effect of allele dosage (allelic tests), recessive action, dominant action, additive+dominant (genotypic tests). Thus, we used the additive dosage model although in those SNPs there was not statistically significant support for the chosen model to be the best.

Six out of eight polymorphisms exhibited allelic association with MS, with nominal P-values ranging from 0.0016 to 0.033 (Table 1). All of them also showed significant association when genotypes instead of alleles and the Cochran-Armitage trend test where used (P values from 0.0016 to 0.033, Table S2). The Cochran-Armitage trend test has better power for near-additive risks models [31] than the Fisher exact test.

rs1570538 has been shown to be weakly associated with multiple sclerosis in our previous study of *IL2RA*/MS in a cohort of 346 cases and 413 controls (allelic P-value = 0.04) [10] and showed here increased statistical significance with our actual extended cohort (allelic association P = 0.033; OR for minor allele = 0.86, 95% CI, 0.75–0.99; genotype association (Cochran-Armitage trend test P = 0.033). We did not find any statistically significant evidence for MS association with either rs41295061 nor for rs12722489 by using the Cochran-Armitage trend test (P = 0.279 and P = 0.133 for rs12722489 and rs41295061 respectively) or the Fisher exact test in genotypes (P = 0.501 and P = 0.243 for rs12722489 and rs41295061 respectively) and alleles (P = 0.278 and P = 0.135 for SNPs rs12722489 and rs41295061 respectively). The strongest association signals in this study were observed for rs7090530 and rs35285258 which, both allelic and genotype association, survived after correction for multiple tests by Bonferroni (Table S2). For the rs7090530, the data revealed a significant over-representation of the C allele among cases compared with controls (OR = 1.23, 95% C.I. = 1.07–1.41, P = 0.003). Very similar data were obtained for the T allele of rs35285258 (OR = 1.26, 95% C.I. = 1.09–1.46, P = 0.0016).

As observed in Fig. 1, rs7090530 and rs35285258 are in high LD with a pairwise r^2^ = 0,53. rs1570538, rs2104286, rs10795791 and rs4147359, are in partial LD ranging from 0.29 to 0.09 with rs35285258. rs12722489 and rs41295061, negative for association with MS in this study, were in very low LD with rs35285258 (r^2^ = 0.06 and 0.033, respectively). Conditioned to rs35285258, rs7090530 did not add any significant association, neither did the other SNPs (P-values 0.2377, 0.1718, 0.5885, 0.3112, 0.4517, 0.2342 and 0.3116 for the seven SNPs respectively). Thus, the rs7090530 association with MS could be explained by its strong LD with rs35285258.

## Discussion

The aim of this study was to determine whether there is a common origin of the association of certain polymorphisms in the region of the *IL2RA* gene in MS and T1D. The fine mapping of the *IL2RA* region in T1D has located two ancestrally distinct causal alleles that are marked by two independent groups of SNPs at the first intron and the 5′ region of the *IL2RA* gene [13]. The genome wide association study of MS showed association with two SNPs (rs2104286 and rs12722489) [8] located at the 5′ region of *IL2RA* [10], that were not in linkage disequilibrium with those most associated with T1D [13]. The 3′ UTR polymorphism, rs1570538, which has been shown to be weakly associated with MS in our previous study [13] is replicated here in a larger cohort showing increased statistical significance. This could be due to the LD that keeps with rs35285258 (r^2^ 0.11) and shows a haplotype-specific effect.

The analysis of polymorphisms in this study reflects the existence of a heterogeneous association between T1D and MS that suggest different immunopathological mechanisms. It is notable that rs41295061, the most associated with T1D [13], was not associated with MS in this study.

rs35285258, belonging to the other independent group of SNPs associated with T1D [13] showed the maximal association in this study. However, the rs35285258 risk allele is C in the T1D study [13] while it was T in our analysis of MS. This is also the case for the rs4147359 and rs7090530 whose risk alleles were the contrary to those observed for T1D [13]. This type of observation has also been described for the *FCRL-3* gene, encoding a member of the Fc receptor-like family, specifically the C allele of FCRL3_3 variant has been associated with susceptibility to several autoimmune diseases [26] but showed to be protective for MS [27], [28], and Addison's disease [32].

We replicated the association of only one (rs2104286) of the two SNPs identified in the GWA study of MS [8]. Moreover SNPs rs1570538, rs2104286, rs12722489, previously associated with MS, have lower levels of statistical significance than SNPs rs10795791, rs4147359, rs7090530, and rs35285258 from the T1D study when typed in MS. This may be due to that the MS associated SNPs come from a non exhaustive study of the locus and they capture the causal SNP with low LD.

It is unknown whether there is any causal polymorphism in the SNPs analyzed here and whether it can affect the level of expression of the *IL2RA* product because none of these polymorphisms are located in known regulatory regions. rs4147359 G allele and the T allele of rs10795791 create a putative recognition site for GATA-1 and GATA-2 transcription factors, respectively. Although these two SNPs did not survive the Bonferroni correction and had no effect conditioned on rs35285258, they showed a significant haplotype-specific effect (data not shown). The A allele of rs7090530 disrupted a putative CpG dinucleotide, as it does the SNP rs11597367 described in the T1D study [13], located at the 5′ region of the IL2RA at position 6147540 and in total LD (r^2^ = 1) with rs35285258. Methylation of the CpG dinucleotides could be important for gene transcription regulation as it has been demonstrated for the IL2 gene [33]

The T1D-risk alleles have been associated with reduced soluble IL2R alpha concentrations. Thus, as the MS susceptibility alleles correspond with the contrary ones to those associated in the T1D study, we assume that the MS risk alleles might be associated with the high concentration phenotype of soluble IL2RA protein. Several aspects of the immune response could also be affected in an opposite fashion to those in the T1D study, for instance, the activity and functioning of activated Th1 and T regulatory cells (CD4+CD25+) but at the moment, the relevance and the role of this potential phenotypic data in the pathogenesis of MS is unknown.

In summary, our results replicate and extend the association found in the *IL2RA* gene region with MS and reveal differences in the polymorphisms and risk alleles associated with T1D which may reflect distinct roles that such gene variants may have in these two pathologies.

## Supporting Information

Table S1 Test to evaluate whether the best model is statistically significant.
(0.03 MB DOC)

Table S2 Genotype distribution for 8 IL2RA SNPs in MS cases and healthy controls and P values for Fisher exact test and Cochran-Armitage trend test performed in genotypes.
(0.03 MB DOC)
