# Supplementary material for: IL2RA/CD25 Gene Polymorphisms: Uneven Association with Multiple Sclerosis (MS) and Type 1 Diabetes (T1D)
Source: PLoS One. 2009 Jan 6;4(1):e4137. doi: 10.1371/journal.pone.0004137 (PMC2607550; doi:10.1371/journal.pone.0004137)
Supplement: Table S1 — Test to evaluate whether the best model is statistically significant. (0.03 MB DOC) [file pone.0004137.s001.doc]

Table S1. Test to evaluate whether the best model is statistically significant.

| Rs (SNP no.) | P value (best model)^a^ | Best model | P-value (permutation test)^b^ |
| --- | --- | --- | --- |
| rs1570538 (SNP1) | 0.0091 | recessive | 0.0190 |
| rs2104286 (SNP2) | 0.1718 | additive | 0.0370 |
| rs12722489 (SNP3) | 0.2895 | additive | 0.4625 |
| rs10795791 (SNP4) | 0.0289 | additive | 0.0709 |
| rs4147359 (SNP5) | 0.0237 | additive | 0.0530 |
| rs7090530 (SNP6) | 0.0035 | additive | 0.0120 |
| rs41295061 (SNP7) | 0.1446 | additive | 0.2018 |
| rs35285258 (SNP8) | 0.0016 | additive | 0.0050 |

(a) P-values for the best model among additive (allelic), recessive, dominant or additive+dominant (genotypic).

(b) P-values for the permutation test performed to evaluate whether the best model is statistically significant.
