# Supplementary material for: IL2RA/CD25 Gene Polymorphisms: Uneven Association with Multiple Sclerosis (MS) and Type 1 Diabetes (T1D)
Source: PLoS One. 2009 Jan 6;4(1):e4137. doi: 10.1371/journal.pone.0004137 (PMC2607550; doi:10.1371/journal.pone.0004137)
Supplement: Table S2 — Genotype distribution for 8 IL2RA SNPs in MS cases and healthy controls and P values for Fisher exact test and Cochran-Armitage trend test performed in genotypes. (0.03 MB DOC) [file pone.0004137.s002.doc]

Table S2

Genotype distribution for 8 *IL2RA* SNPs in MS cases and healthy controls and P values for Fisher exact test and Cochran-Armitage trend test performed in genotypes.

SNP rs No

Genoypes CASES CONTROLS P P P

Fisher Trend Bonferroni

exact test corrected

test

rs1570538 1

TT 203 (0,26) 203 (0,25) 0.030 0.033 0.24

CT 396 (0,52) 393 (0,48)

CC 169 (0,22) 229 (0,28)

rs2104286 2

TT 508 (0,67) 574 (0,63) 0.044 0.017 0.32

CT 224 (0,3) 296 (0,32)

CC 21 (0,03) 42 (0,05)

­­­­­­­­­­­­­­­

rs12722489 3

GG 538 (0,78) 518 (0,76) 0.501 0.279 -

AG 140 (0,2) 150 (0,022)

AA 8 (0,012) 12 (0,018)
­­­­­­­­­­­­­­­­­­

rs10795791 4

AA 306 (0,42) 342 (0,38) 0.072 0.029 -

AG 336 (0,45) 418 (0,46)

GG 95 (0,13) 151 (0,16)

rs4147359 5

GG 404 (0,525) 429 (0,477) 0.061 0.023 -

AG 312 (0,41) 379 (0,42)

AA 54 (0,07) 91 (0,1)

rs7090530 6

AA 176 (0,230) 269 (0,29) 0.010 0.003 0.08

AC 395 (0,52) 464 (0,50)

CC 192 (0,252) 192 (0,208)

rs41295061 7

CC 678 (0,89) 753 (0,870) 0.243 0.133 -

AC 82 (0,11) 109 (0,13)

AA 1 (0,001) 4 (0,005)

rs35285258 8

CC 288 (0,38) 406 (0,46) 0.006 0.0016 0.048

CT 360 (0,48) 384 (0,43)

TT 101 (0,14) 93 (0,1)
